# Probiotics for prevention of necrotizing enterocolitis in preterm infants: systematic review and meta-analysis

**DOI:** 10.1186/s13052-015-0199-2

**Published:** 2015-11-14

**Authors:** Arianna Aceti, Davide Gori, Giovanni Barone, Maria Luisa Callegari, Antonio Di Mauro, Maria Pia Fantini, Flavia Indrio, Luca Maggio, Fabio Meneghin, Lorenzo Morelli, Gianvincenzo Zuccotti, Luigi Corvaglia, on behalf of the Italian Society of Neonatology

**Affiliations:** Neonatal Intensive Care Unit, Department of Medical and Surgical Sciences (DIMEC), University of Bologna, S.Orsola-Malpighi Hospital, Bologna, Italy; Department of Biomedical and Neuromotor Sciences (DIBINEM), University of Bologna, Bologna, Italy; Neonatal Unit, Catholic University, Rome, Italy; Institute of Microbiology, UCSC, Piacenza, Italy; Department of Pediatrics, Aldo Moro University, Bari, Italy; Department of Pediatrics, University of Milan, Luigi Sacco Hospital, Milan, Italy

**Keywords:** Probiotics, Newborn, Necrotizing enterocolitis, Meta-analysis

## Abstract

Necrotizing enterocolitis (NEC) affects predominantly preterm infants, who have specific risk factors leading to intestinal *dysbiosis*. Manipulations of gut microbiota through probiotics have the potential to prevent NEC.

The aim of this systematic review and meta-analysis was to evaluate the effect of probiotics for NEC prevention in preterm infants, with a focus on specific strains, microbiological strength of currently available studies, and high-risk populations.

PubMed and the Cochrane Library were searched for trials published within 4th February 2015. Randomized-controlled trials reporting on NEC and involving preterm infants who were given probiotics in the first month of life were included in the systematic review.

Twenty-six studies were suitable for inclusion in the meta-analysis.

Data about study design, population, intervention and outcome were extracted and summarized independently by two observers. Study quality and quality of evidence were also evaluated.

Fixed-effects models were used and random-effects models where significant heterogeneity was present. Subgroup analyses were performed to explore sources of heterogeneity among studies. Results were expresses as risk ratio (RR) with 95 % confidence interval (CI).

The main outcome was incidence of NEC stage ≥2 according to Bell’s criteria.

Probiotics prevented NEC in preterm infants (RR 0.47 [95 % CI 0.36–0.60], *p* < 0.00001). Strain-specific sub-meta-analyses showed a significant effect for *Bifidobacteria* (RR 0.24 [95 % CI 0.10–0.54], *p* = 0.0006) and for probiotic mixtures (RR 0.39 [95 % CI 0.27–0.56], *p* < 0.00001). Probiotics prevented NEC in very-low-birth-weight infants (RR 0.48 [95 % CI 0.37–0.62], *p* < 0.00001); there were insufficient data for extremely-low-birth-weight infants. The majority of studies presented severe or moderate microbiological flaws.

Probiotics had an overall preventive effect on NEC in preterm infants. However, there are still insufficient data on the specific probiotic strain to be used and on the effect of probiotics in high-risk populations such as extremely-low-birth-weight infants, before a widespread use of these products can be recommended.

## Background

Necrotizing enterocolitis (NEC), which is one of the most devastating neonatal diseases, has become a priority for research [1]. Despite great advances in neonatal care, the morbidity, mortality and health-care costs directly related to the disease are substantial: during hospital stay, the economic burden of NEC in the United States has been estimated as high as several billions USD per year, which is approximately 20 % of the costs for Neonatal Intensive Care Units in the country; furthermore, this estimate is likely to be much higher when the costs of long-term care of survivors are taken into account [2].

NEC is a multifactorial disease: prematurity is a well-recognized risk factor, and approximately 90 % of the infants who develop NEC are born preterm [3]. This is probably due to specific comorbidities of prematurity, such as immunodeficiency, use of broad-spectrum antimicrobials, delayed enteral feeding and low availability of human milk.

Recently, research has focused on the role of gut microbiota and its manipulations, such as the use of probiotics, on disease and health status. Probiotics are live–microorganisms which, when ingested in adequate amounts, confer a health-benefit to the host through an interaction with gut microbiota [4]. The intestinal microbiota undergoes dynamic changes during childhood. Gut colonization in preterm infants occurs differently than in healthy term newborns [5], and preterm infants frequently have delayed and aberrant acquisition of the “normal” digestive flora. Recent studies performed in preterm foetuses and infants demonstrated that amniotic fluid and meconium are not sterile, suggesting an intrauterine origin of gut microbiota [6, 7]; after birth, the preterm infant’s immature intestine is exposed to an unique environment and to several iatrogenic manipulations, including the use of broad-spectrum antibiotics. The subsequent intestinal *dysbiosis* is recognized as a risk factor for NEC: actually, it has been shown that preterm infants with NEC have reduced bacterial gut diversity and different bacterial strains compared to healthy controls [8]. In this perspective, provision of probiotics to preterm infants has the potential to “normalize” the abnormal colonization pattern, thus preventing the occurrence of the disease [9].

The use of probiotics for the prevention of NEC in preterm infants has been extensively investigated in many randomized-controlled trials, whose results have been summarized in several systematic-reviews and meta-analyses [10, 11]. The authors of these meta-analyses, which show that probiotics reduce NEC and mortality in preterm infants, strongly encourage a change in practice, promoting a widespread use of probiotics in this population [11], and also claim that withholding probiotics from high-risk neonates would be almost unethical [10]. However, the position of the American Academy of Paediatrics is more cautious, highlighting the need for more studies to address unanswered questions on the amount and specificity of which probiotic or mixture of probiotics should be used [12]. In addition, a recent systematic review, which analyzed the level of evidence of randomized-controlled trials on probiotics in preterm infants, concluded that there is still insufficient evidence to recommend routine probiotics use, but also that present data are encouraging and justify further research on specific probiotic products [13].

Actually, the beneficial effects of probiotics appear to be strain-specific, and pooling data from studies using different strains can result in misleading conclusions [14]. Furthermore, currently available studies often lack specificity in reporting correct identification of probiotic strain [15], dosage regimen and duration, and gut colonization, which are all fundamental to assess the ability of a probiotic to confer a health benefit to the host [16].

The aim of this meta-analysis is thus to evaluate in detail the effect of probiotics for the prevention of NEC in preterm infants, with a focus on specific strains, on microbiological strength of currently available studies, and on high-risk populations.

## Methods

### Literature search

The study protocol was designed jointly by the members of the Task Force on Probiotics of the Italian Society of Neonatology.

A systematic review of published studies reporting the use of probiotics for the prevention of NEC in preterm infants was performed, following PRISMA guidelines [17].

Criteria for inclusion in the meta-analysis were the following: randomized and quasi-randomized controlled trials involving preterm infants (gestational age <37 weeks) and reporting on NEC (any stage, according to modified Bell staging criteria [18, 19]); enteral administration of any probiotic starting within one month of age, compared to placebo or no treatment. Being the search strategy focused specifically on NEC, data on different outcomes, such as sepsis or mortality, which were reported in the studies retrieved by the literature search, were not evaluated by meta-analysis.

**Fig. 1:**
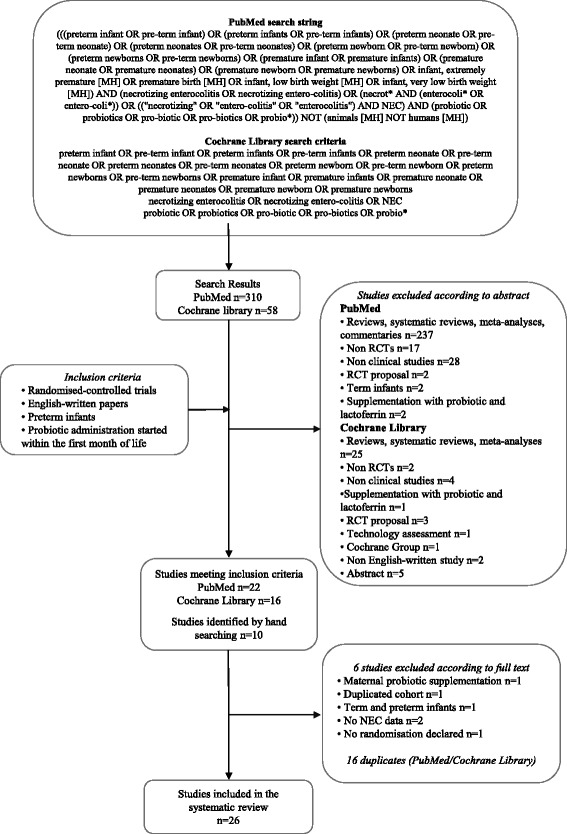
Flow chart showing the search strategy and search results. The relevant number of papers at each point is given

A search was conducted in PubMed (http://www.ncbi.nlm.nih.gov/pubmed/) for studies published before 4th February 2015, using the search string reported in Fig. 1. This string was built up combining all the terms related to NEC and probiotics, using PubMed MeSH terms and free-text words and their combinations through the most proper Boolean operators, in order to be as comprehensive as possible. Similar criteria were used for searching the Cochrane Library. The review was limited to studies written in English and involving human subjects.

The search was conducted by AA and LC: relevant studies were identified from the abstract, and reference lists of papers retrieved were searched for additional studies. “Snowballing” technique was also used [20].

### Data extraction and meta-analysis

Study details, including study population, characteristics of the intervention, use of placebo, and outcome, were assessed independently by AA and LC, and checked by DG. Study quality was evaluated independently using the risk of bias tool as proposed by the Cochrane collaboration (Chapter 8 of the Cochrane Handbook of Systematic Reviews) [21]. In addition, an assessment of the body of evidence using the GRADE working group approach was used in order to grade the quality of evidence. The evaluation was carried out following the Chapter 12 of the Cochrane Handbook [21] and classifying the evidence as high, moderate, low and very low (as suggested by the GRADE Working Group) [22].

The association between probiotic use and NEC was evaluated by meta-analyses, conducted by AA and DG, using the RevMan software (version 5.3.5; downloaded from the Cochrane website: http://tech.cochrane.org/revman/download). Risk ratio (RR) was calculated using the Mantel-Haenszel method, and reported with 95 % confidence interval (CI).

The following sub-meta-analyses were also performed, in order to evaluate the effect of probiotics:

- in specific subgroups of patients (very-low-birth-weight [VLBW] infants);
- in surgical NEC;
- according to NEC incidence in different populations: the incidence of NEC stage ≥2 in the control population was used as a reference, because only a minority of studies reported NEC incidence in the general population. Studies were arbitrarily divided into three groups defined as “low-risk” (NEC incidence <5 %), “medium-risk” (incidence 5–10 %), and “high-risk” (incidence >10 %);
- according to probiotic strain: studies were divided according to the specific probiotic strain used, and were considered as suitable for inclusion in the sub-meta-analyses when the same probiotic strain was used in at least two studies. Studies which used a probiotic mixture were considered together.

Microbiological quality of all the studies was evaluated by MLC and LM. Studies were defined as having severe, moderate or minor microbiological flaws according to the evaluation of proper strain identification and microbiological assessment. Specifically, the lack of proper strain identification was considered as a severe flaw; the lack of microbiological assessment regarding the probiotic persistence in stools was considered as a moderate flaw, whereas a low flaw was defined when the presence of the probiotic in stools was evaluated by indirect approaches such as the quantification of its species belonging.

A fixed-effect model was used for the analyses. Heterogeneity was measured using the I^2^ test. If significant heterogeneity was present (*p* < 0.05 from the *χ*^2^ test), a random-effects model was used [23]. The random-effects model was also used when heterogeneity was not significant but the number of studies was ≤ 5, because the test for heterogeneity is known to have low power when the number of studies is small [24].

Forest plots were used to illustrate results from meta-analyses, and funnel plots to investigate bias.

The online version of GraphPad Quickcalcs software was used to calculate number needed to treat (NNT).

## Results

### Literature search

Three-hundred-sixty-eight papers were identified through the literature search (310 through PubMed and 58 through the Cochrane Library). Thirty-eight studies met the inclusion criteria: 22 were identified through the PubMed search [25–48] and 16 through the Cochrane Library search [25, 26, 28–32, 34–37, 40–43, 45]. Ten additional papers were identified from the reference lists of included studies [49–58]. Of these 47 studies, 16 were excluded, as they were duplicates retrieved both by PubMed and Cochrane Library search. Six additional studies were excluded after examining the full-texts: one study reported maternal probiotic supplementation during pregnancy [29], one cohort was reported twice [31], one study included both term and preterm infants [36], two studies did not report NEC data [55, 56], and in one study randomization was not declared [54].

Twenty-six studies were suitable for inclusion in the meta-analysis [10, 25–28, 30, 32–35, 37–42, 44, 46, 48–53, 58]. A description of included studies is provided in Table 1; excluded studies are described in Table 2.

**Table 1:** Studies included in the systematic review and meta-analysis

Author, year	Study details	Study population	Intervention	Type of milk	Placebo
			- Strain		
			- Dose (D)		
			- Start of treatment (S)		
			- End of treatment (E)		
Al-Hosni, 2012 [33]	P	Preterm infants with BW 501–1000 g, appropriate for gestational age, and ≤ 14 days of age at time of feeding initiation	*Lactobacillus rhamnosus GG LGG*	Non specified	Extra milk
	DB		*Bifidobacterium infantis*		
	R		D: 0.5 × 10^9^ CFU each probiotic, OD		
	C		S: first enteral feeding		
	Multic.		E: discharge or until 34 w postmenstrual age		
Bin-Nun, 2005 [40]	P	Preterm infants with BW < 1500 g, who began enteral feeding on a weekday	*Bifidobacterium infantis*	OMM, PFM	HM or FM
	B		*Streptococcus thermophilus*		
	R		*Bifidobacterium bifidus*		
	C		D: 0.35 × 10^9^ CFU each probiotic, OD		
			S: start of enteral feeding		
			E: 36 w postconceptual age		
Braga, 2011 [35]	P	Inborn infants with BW 750–1499 g	*Lactobacillus casei*	HM	Extra HM
	DB		*Bifidobacterium breve*		
	R		D: 3.5 × 10^7^ CFU to 3.5 × 10^9^ CFU OD		
	C		S: day 2		
			E: day 30, NEC diagnosis, discharge, death, whichever occurred first		
Costalos, 2003 [49]	P	GA 28–32 w	*Saccharomyces boulardii*	PFM	MDX
	R	No major GI problem	D: 1 × 10^9^ CFU BD		
	C	Not receiving antibiotics	S: non-specified		
		Not receiving breast milk	Median duration of probiotic supplementation: 30 days		
Dani, 2002 [42]	P	Infants with GA < 33 w or BW < 1500 g	*Lactobacillus rhamnosus GG*	OMM, DM or FM	MDX
	DB		D: 6 × 10^9^ CFU OD		
	R		S: first feed		
	C		E: discharge		
	Multic.				
Demirel, 2013 [28]	P	Preterm infants with GA ≤ 32 w and BW ≤ 1500 g, who survived to feed enterally	*Saccharomyces boulardii*	HM, FM	None
	B		D: 5 × 10^9^ CFU OD		
	R		S: first feed		
	C		E: discharge		
Dilli, 2015 [44]	P	Preterm infants with GA <32 weeks and BW <1500 g, born at or transferred to the NICU within the first week of life and fed enterally before inclusion	*Bifidobacterium*	HM, FM	MDX powder
	DB		*Lactis*		
	R		D: 5 × 10^9^ CFU		
	C		S: beyond d7 after birth		
	Multic.		E: death or discharge (max 8 weeks)		
Fernández-Carrocera, 2013 [32]	P	Preterm infants with BW < 1500 g	*Lactobacillus acidophilus* 1 CFU/g	OMM, PFM	None
	DB	Infants with NEC IA and IB were excluded	*Lactobacillus rhamnosus* 4.4 × 10^8^ CFU/g		
	R		*Lactobacillus casei* 1 × 10^9^ CFU/g		
	C		*Lactobacillus plantarum* 1.76 × 10^8^ CFU/g		
			*Bifidobacterium infantis* 2.76 × 10^7^ CFU/g		
			*Streptococcus thermophilus* 6.6 × 10^5^ CFU/g		
			Total D: 1 g powder OD		
			S: start of enteral feeding		
			E: non-specified		
Jacobs, 2013 [26]	P	Preterm infants with GA <32 w and BW < 1500 g	*Bifidobacterium infantis BB-02* 300 CFU × 10^6^	HM, FM	MDX powder
	DB		*Streptococcus thermophilus Th-4* 350 CFU × 10^6^		
	R		*Bifidobacterium lactis BB-12* 350 CFU × 10^6^		
	C		Total D: 1 × 10^9^ CFU × 1.5 g maltodextrin powder OD		
	Multic.		S: enteral feed ≥ 1 ml every 4 h		
			E: discharge or term corrected age		
Kitajima, 1997 [52]	P	Preterm infants with BW < 1500 g	*Bifidobacterium breve YIT4010*	OMM, FM after full enteral feeding had been reached	Distilled water
	R		D: 0.5 × 10^9^ CFU OD		
	C		S: within 24 h of life		
			Duration of probiotic supplementation: 28 days		
Lin, 2005	P	Infants with BW < 1500 g, who started to feed enterally and survived beyond day 7	*Lactobacillus acidophilus*	OMM, DM	None
	M		*Bifidobacterium infantis*		
	R		D: ≥ 10^6^ CFU each probiotic (=125 mg/kg), BD		
	C		S: start of enteral feeding		
			E: discharge		
Lin, 2008	P	Preterm infants with GA < 34 w and BW ≤ 1500 g, who survived to feed enterally	*Lactobacillus acidophilus NCDO 1746*	HM, FM	None
	B		*Bifidobacterium bifidum NCDO 1453*		
	R		D: 1 × 10^9^ CFU each probiotic (=125 mg/kg) BD		
	C		S: day 2 of age		
	Multic.		Duration: 6 weeks		
Manzoni, 2006 [37]	P	Infants with BW < 1500 g, ≥ 3 days of life, who started enteral feeding with HM	*Lactobacillus rhamnosus LGG*	OMM, DM	None
	DB		D: 6 × 10^9^ CFU/day		
	R		S: day 3 of life		
	C		E: end of the 6th week or discharge		
Mihatsch, 2010 [43]	P	Preterm infants with GA < 30 w and BW ≤ 1500 g	*Bifidobacterium lactis BB12*	HM, PFM	Indistinguishable powder
	R		D: 2 × 10^9^ CFU/kg 6 times a day		
	C		S: start of enteral feeding		
			E: non-specified		
Mohan, 2006 [53]	P	Preterm infants (GA < 37 w)	*Bifidobacterium lactis BB12*	FM	Not stated
	DB		D: 1.6 × 10^9^ CFU on day 1 to 3, and 4.8 × 10^9^ CFU from day 4 onwards		
	R		S: first day of life		
	C		Duration: 21 days		
Oncel, 2013 [25]	P	Preterm infants with GA ≤ 32 w and BW ≤ 1500 g, who survived to feed enterally	*Lactobacillus reuteri DSM 17938*	HM, FM	Oil base
	DB		D: 1 × 10^8^ CFU OD		
	R		S: first feed		
	C		E: death or discharge		
Patole, 2014 [45]	P	Preterm infants with GA < 33 w and BW < 1500 g	*Bifidobacterium breve* M16-V	HM, FM	Dextrin
	DB		D: 3 × 10^9^ CFU OD (1.5 × 109 CFU OD for newborns ≤ 27 w until they reached 50 ml/kg/day enteral feeds)		
	R		S: start of enteral feed		
	C		E: corrected age of 37 w		
Rojas, 2012 [30]	P	Preterm infants with BW ≤ 2000 g, hemodynamically stable, ≤ 48 h of age (regardless start of enteral feeding)	*Lactobacillus reuteri DSM 17938*	HM, FM	Oil base
	DB		D: 1 × 10^8^ CFU OD		
	R		S: age ≤ 48 h		
	C		E: death or discharge		
	Multic.				
Rougé, 2009 [50]	P	Preterm infants with GA < 32 w and BW < 1500 g, ≤ 2 w of age, without any disease other than those linked to prematurity, who started enteral feeding before inclusion	*Bifidobacterium longum BB536*	OMM, DM or PFM	MDX
	DB		*Lactobacillus rhamnosus GG BB536-LGG*		
	R		Total D: 1 × 10^8^ CFU/day		
	C		S: start of enteral feeding		
	Bic.		E: discharge		
Roy, 2014 [58]	P	Preterm infants (GA < 37w) and BW < 2500 g, with stable enteral feeding within 72 h of birth	*Lactobacillus acidophilus* 1.25 × 10^9^ CFU × 1 g	HM	Sterile water
	DB		*B. longum* 0.125 × 10^9^ CFU × 1 g		
	R		*B. bifidum* 0.125 × 10^9^ CFU × 1 g		
	C		*B. lactis* 1 × 10^9^ CFU × 1 g		
			D: half a 1 g sachet		
			S: from 72 h of life		
			E: after 6 w or at discharge		
Saengtawesin, 2014 [48]	P	Preterm infants with GA ≤ 34 w and BW ≤ 1500 g	*Lactobacillus acidophilus* 1 × 10^9^ CFU	HM, PFM	None
	R		*Bifidobacterium*		
	C		*bifidum* 1 × 10^9^ CFU		
			D: 125 mg/kg BD		
			S: start of feeding		
			E: 6 w of age or discharge.		
Samanta, 2009	P	Preterm infants with GA < 32 w and BW < 1500 g, who started enteral feeding and survived beyond 48 h of age	*Bifidobacterium infantis*	HM	None
	DB		*Bifidobacterium bifidum*		
	R		*Bifidobacterium longum*		
	C		*Lactobacillus acidophilus*		
			D: 2.5 × 10^9^ CFU each probiotic, BD		
			S: start of enteral feeding		
			E: discharge		
Sari, 2011 [34]	P	Preterm infants with GA < 32 w or BW < 1500 g, who survived to feed enterally	*Lactobacillus sporogenes*	HM, FM	None
	B		D: 0.35 × 10^9^ CFU OD		
	R		S: first feed		
	C		E: discharge		
Serce, 2013 [27]	P	Preterm infants with GA ≤ 32 w and BW ≤ 1500 g, who survived to feed enterally	*Saccharomyces boulardii*	HM, FM	Distilled water
	M		D: 0.5 × 10^9^ CFU/kg BD		
	R		S: non-specified		
	C		E: non-specified		
Stratiki, 2007 [39]	P	Preterm infants with GA 27–32 w, formula-fed, without major congenital anomalies	*Bifidobacterium lactis*	FM	None
	B		D: 2 × 10^7^ CFU/g of milk powder		
	R		S: start of enteral feeding		
	C		E: non-specified		
Totsu, 2014 [46]	P	Infants with BW < 1500 g	*Bifidobacterium bifidum*	HM, FM	Dextrin
	DB		D: 2.5 × 10^9^ CFU, divided in two doses		
	CLR		S: within 48 h after birth		
	C		E: body weight 2000 g		
	Multic.				

*P* prospective, *DB* double-blinded, *R* randomized, *C* controlled, *Multic* multicentric, *B* blinded, *M* masked, *Bic* bicentric, *BW* birth weight, *GA* gestational age, *NEC* necrotizing enterocolitis, *HM* human milk, *CFU* colony forming unit, *OD* once daily, *BD* twice daily, *OMM* own mother’s milk, *PFM* preterm formula, *DM* donor milk, *FM* formula, *MDX* maltodextrin

**Table 2:** Studies excluded from the systematic review and meta-analysis

Author, year	Study summary	Reason for exclusion
Awad, 2010	Living vs. killed *Lactobacillus acidophilus* vs. placebo given to neonates admitted to the study NICU	Term and preterm infants included
Benor, 2014	*Lactobacillus acidophilus* and *Bifidobacteria lactis* vs. placebo given to mothers of VLBW infants	Maternal probiotic supplementation
Li, 2004	*Bifidobacterium breve* given to LBW infants	Randomization not declared
Millar, 1993	*Lactobacillus GG* given to preterm infants with GA < 33 w	No NEC data
Reuman, 1986	Formula containing lactobacilli vs. placebo given to preterm infants	No NEC data
Sari, 2012	*Lactobacillus sporogenes* given to preterm infants with GA < 32 w or BW < 1500 g, who survived to feed enterally	Duplicate population (Sari, 2011 [34])

*NICU* neonatal intensive care unit, *VLBW* very low birth weight, *LBW* low birth weight, *GA* gestational age, *NEC* necrotizing enterocolitis, *BW* birth weight

All the studies reported NEC data in a form suitable for meta-analysis, except one [53], for which data included in a previous Cochrane review were used [59]. For each study, NEC rate in the probiotic and in the placebo/control group is reported in Table 3. For the purpose of the meta-analysis, data on NEC stage ≥2 were used.

**Table 3:** Incidence of necrotizing enterocolitis in infants treated with probiotics and in controls

Author, year	Previous NEC rate	Number of subjects	NEC in probiotic group	NEC in control group
Al-Hosni, 2012 [33]	Not stated	50 probiotic	3/50 any stage	4/51 any stage
		51 control	1/50 stage 1	2/51 stage 1
			0/50 stage 2	0/51 stage 2
			2/50 stage 3	2/51 stage 3
Bin-Nun, 2005 [40]	15 %	72 probiotic	3/72 any stage	12/73 any stage
		73 control	1/72 stage ≥2	10/73 stage ≥2
			1/72 stage 2	7/73 stage 2
			0/72 stage 3	3/73 stage 3
Braga, 2011 [35]	10 %	119 probiotic	0/119 stage ≥2	4/112 stage ≥2
		112 placebo		
Costalos, 2003 [49]	Not stated	51 probiotic	5/51 any stage	6/36 any stage
		36 placebo		
Dani, 2002 [42]	Not stated	295 probiotic	4/295 stage ≥2	8/290 stage ≥2
		290 placebo		
Demirel, 2013 [28]	32 %	135 probiotic	6/135 stage ≥2	7/136 stage ≥2
		136 control		
Dilli, 2015 [44]	Not stated	100 probiotic	2/100 stage ≥2	18/100 stage ≥2
		100 placebo		
Fernández-Carrocera, 2013 [32]	20 %	75 probiotic	6/75 stage ≥2	12/75 stage ≥2
		75 placebo		
Jacobs, 2013 [26]	Not stated	548 probiotic	11/548 stage ≥2	24/551 stage ≥2
		551 placebo		
Kitajima, 1997 [52]	Not stated	45 probiotic	0/45 any stage	0/46 any stage
		46 placebo		
Lin, 2005 [41]	Approx. 23 % (NEC or death)	180 probiotic	2/180 stage ≥2	10/187 stage ≥2
		187 control	2/180 stage 2	4/187 stage 2
			0/180 stage 3	6/187 stage 3
Lin, 2008 [37]	Approx.	217 placebo	4/217 any stage	14/217 any stage
		217 control	2/217 stage 2	9/217 stage 2
			2/217 stage 3	5/217 stage 3
Manzoni, 2006 [37]	Not stated	39 probiotic	1/39 any stage	3/41 any stage
		41 control	1/39 stage 2	2/41 stage 2
			0/39 stage 3	1/41 stage 3
Mihatsch, 2010 [43]	Not stated	84 probiotic	2/84 stage ≥2	4/82 stage ≥2
		82 placebo		
Mohan, 2006 [53]	Not stated	21 probiotic	2/37 stage ≥2	1/32 stage ≥2
		17 placebo	Unpublished data, taken from Alfaleh 2011 [58]	Unpublished data, taken from Alfaleh 2011 [58]
Oncel, 2013 [25]	15 %	200 probiotic	8/200 stage ≥2	10/200 stage ≥2
		200 placebo		
Patole, 2014 [45]	Not stated	74 probiotic	0/74 stage ≥2	1/66 stage ≥2
		66 placebo		
Rojas, 2012 [30]	Not stated	372 probiotic	NEC stage ≥2	NEC stage ≥2
		378 placebo	≤1500 g	≤1500 g
			6/176 probiotic	10/184 placebo
			>1500 g	>1500 g
			3/196 probiotic	5/194 placebo
Rougé, 2009 [50]	Not stated	45 probiotic	2/45 any stage	1/49 any stage
		49 placebo		
Roy, 2014 [58]	Not stated	56 probiotic	2/56 any stage	2/56 any stage
		56 placebo		
Saengtawesin, 2014 [48]	Not stated	31 probiotic	1/31 stage ≥2	1/29 stage ≥2
		29 placebo		
Samanta, 2009	Not stated	91 probiotic	5/91 stage ≥2	15/95 stage ≥2
		95 control		
Sari, 2011 [34]	Approx. 32 % (death or NEC)	110 probiotic	6/110 stage ≥2	10/111 stage ≥2
		111 control	4/110 stage 2	7/111 stage 2
			2/110 stage 3	3/111 stage 3
Serce, 2013 [27]	17 %	104 probiotic	7/104 stage ≥2	7/104 stage ≥2
		104 placebo		
Stratiki, 2007 [39]	Not stated	41 probiotic	0/41 stage ≥2	3/34 stage ≥2
		34 control		
Totsu, 2014 [46]	Not stated	153 probiotic	0/153 stage ≥1	0/130 stage ≥1
		130 control		

*NEC* necrotizing enterocolitis

### Probiotics and NEC stage ≥2

**Fig. 2:**
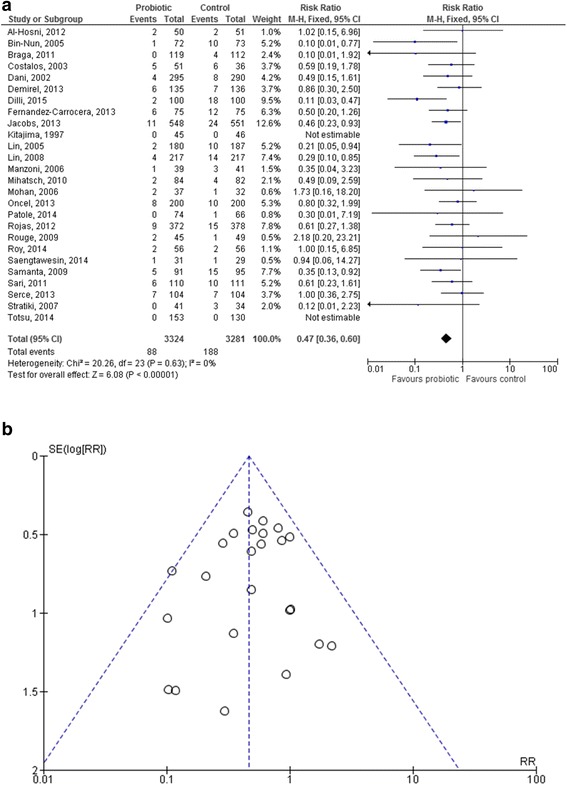
Forest plot (2**a**) and funnel plot (2**b**) of the included studies. The forest plot shows the association between the use of probiotics and necrotizing enterocolitis in the overall population of preterm infants. The funnel plot does not show any clear visual asymmetry. M-H: Mantel-Haenszel method

Data from 6605 infants (3324 in the probiotic group and 3281 in the control group) were analyzed. Fewer infants in the probiotic group developed NEC stage ≥2 compared to infants in the control group (88 [2.65 %] vs. 188 [5.73 %], respectively). The RR was significantly lower in infants treated with probiotics (0.47 [95 % CI 0.36–0.60], *p* < 0.00001; fixed-effect analysis). NNT was 33 (95 % CI 24.7–47.2), which means that 33 infants needed to be treated with probiotics in order to prevent one more case of NEC stage ≥2. Heterogeneity among trials was absent (I^2^ = 0 %, *p* = 0.63; Fig. 2a). The funnel plot did not show any clear asymmetry (Fig. 2b).

### VLBW infants

Twenty-two studies [25–28, 30, 32–35, 37, 38, 40–42, 44–46, 48, 50–52] reported data from 5912 VLBW infants, 2959 in the probiotic and 2953 in the control group. NEC stage ≥2 occurred less frequently in the probiotic group than in controls (82 [2.77 %] infants vs. 174 [5.89 %], respectively), with a RR of 0.48 ([95 % CI 0.37–0.62], *p* < 0.00001; fixed-effect analysis; I^2^ = 0 %, *p* = 0.56). NNT was 33 (95 % CI 24.1–47.9).

### Surgical NEC

Only 6 studies [33, 34, 37, 40, 41, 51] reported separate data for surgical NEC (NEC stage 3), which occurred in 6/668 (0.90 %) infants in the probiotic group and in 20/680 (2.94 %) infants in the control group. The RR for NEC stage 3 was significantly lower in the probiotic group (0.35 [95 % CI 0.16–0.81], *p* = 0.01; fixed-effect analysis; I^2^ = 0 %, *p* = 0.69). NNT was 49 (95 % CI 28.6–170.8).

### NEC incidence

**Fig. 3:**
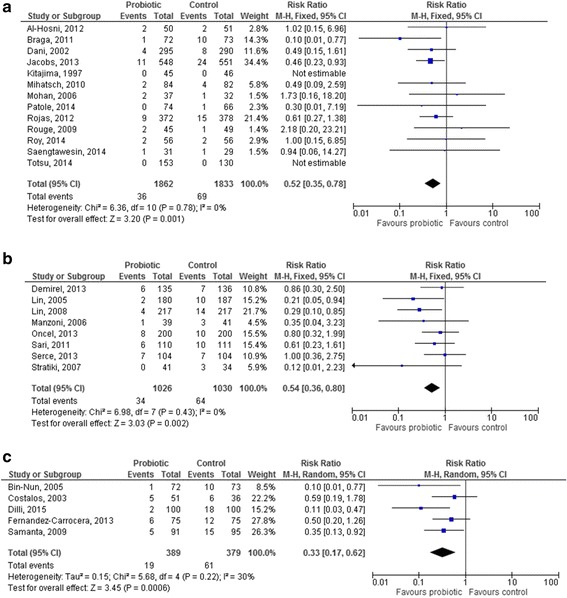
Forest plot showing the association between the use of probiotics and necrotizing enterocolitis (NEC), according to NEC incidence: (3**a**). NEC incidence < 5 %; (3**b**). NEC incidence 5–10 %; (3**c**). NEC incidence >10 %. M-H: Mantel-Haenszel method

NEC incidence in controls was <5 % in 13 studies (Fig. 3a) [26, 30, 33, 35, 42, 43, 45, 46, 48, 50, 52, 53, 58], between 5 and 10 % in 8 studies (Fig. 3b) [25, 27, 28, 34, 37, 39, 41, 51], and >10 % in 5 studies (Fig. 3c) [32, 38, 40, 44, 49].

The RR for NEC stage ≥2 was significantly lower in the probiotic group compared to the control group in all the three populations (RR 0.52 [95 % CI 0.35–0.78], *p* = 0.001; RR 0.54 [95 % CI 0.36–0.80], *p* = 0.002; RR 0.33 [95 % CI 0.17–0.62], *p* = 0.0006, respectively). Heterogeneity was non-significant in all the three sub-analyses.

### Probiotic strain

**Fig. 4:**
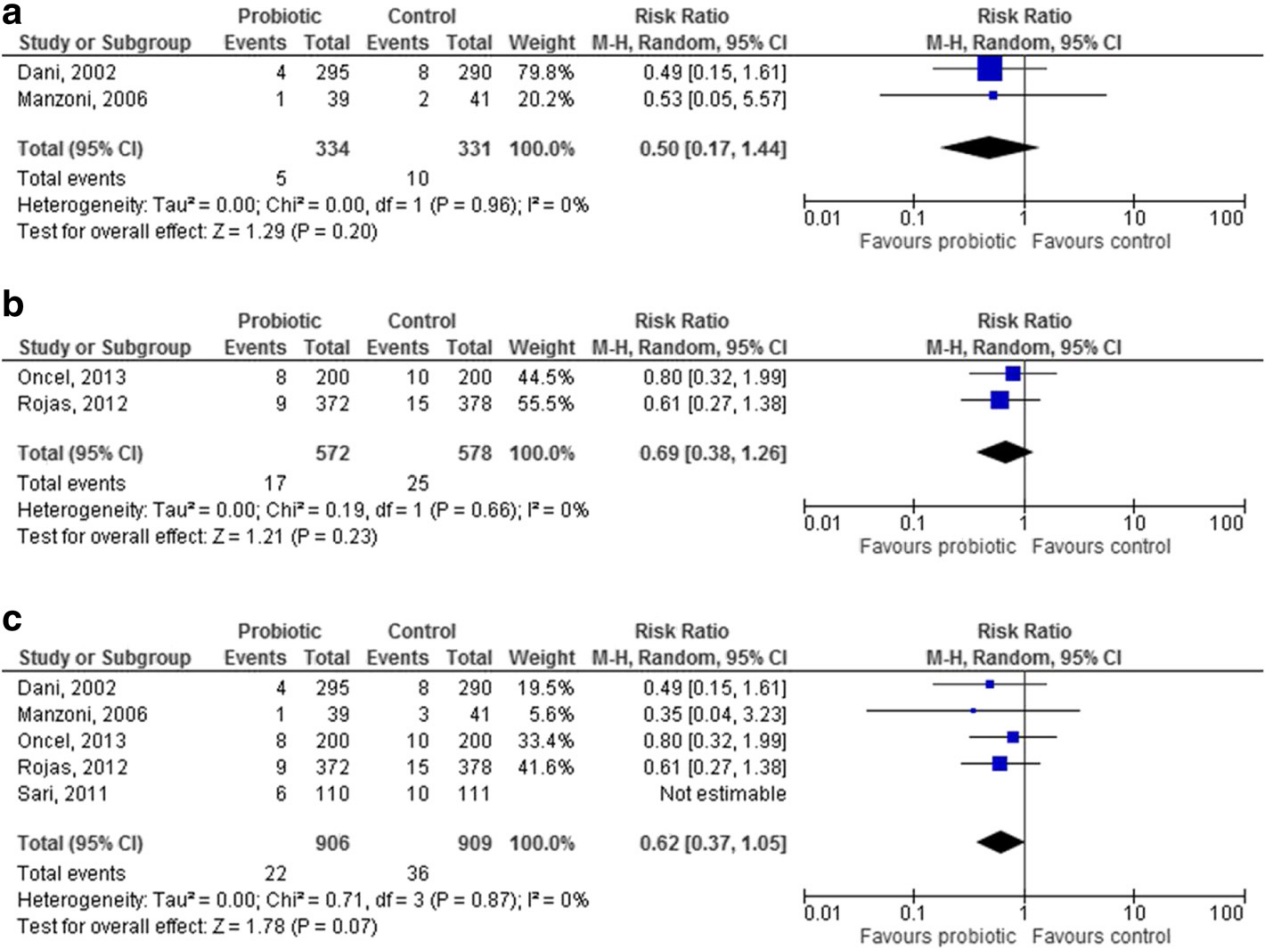
Forest plot showing the association between probiotics and necrotizing enterocolitis in the studies which used a single-strain product containing *Lactobacilli* ((4**a**). *L. reuteri*; (4**b**). *L. GG*; (4**c**). pooled analysis of all the studies using *Lactobacilli*). M-H: Mantel-Haenszel method

*Lactobacillus GG* was used in 2 studies [42, 51] and *Lactobacillus reuteri* in 2 other studies [25, 30]: the effect of these probiotics in reducing NEC was not significant, either for *Lactobacillus GG* and *Lactobacillus reuteri* (RR 0.50 [95 % CI 0.17–1.44], *p* = 0.20 [Fig. 4a], and RR 0.69 [95 % CI 0.38–1.26], *p* = 0.23 [Fig. 4b]). One study used *Lactobacillus sporogenes* [34].

The results of all the studies including *Lactobacilli* were pooled, except for the study by Sari et al. [34]: *Lactobacillus sporogenes* is a species which has not an international recognition, shows characteristics of both *genera Lactobacillus* and *Bacillus*, and its strains should be better classified as *Bacillus coagulans* [60]. Thus, when the results of studies using *Lactobacillus GG* and *reuteri* were pooled, no significant reduction in the RR for NEC in the probiotic group was observed (0.62 [95 % CI 0.37–1.05], *p* = 0.07, Fig. 4c).

**Fig. 5:**
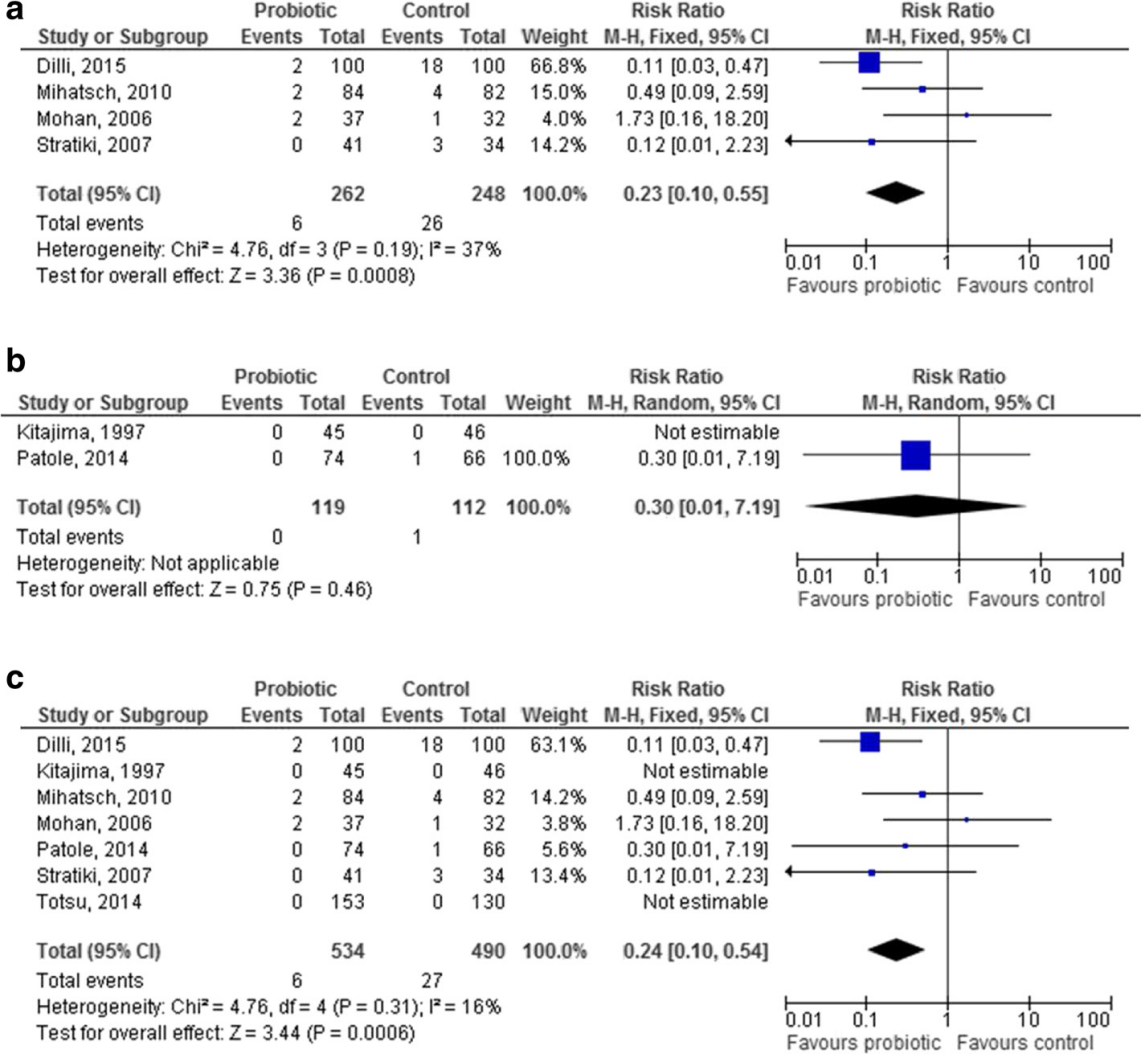
Forest plot showing the association between probiotics and necrotizing enterocolitis in the studies which used a single-strain product containing *Bifidobacteria* ((5**a**). *B. lactis*; (4**b**). *B. breve*; (4**c**). pooled analysis of all the studies using *Bifidobacteria*). M-H: Mantel-Haenszel method

Four studies used *Bifidobacterium lactis* [39, 43, 44, 53], 2 studies *Bifidobacterium breve* [45, 52] and 1 study *Bifidobacterium bifidum* [46]. The use of *Bifidobacterium lactis* resulted in a significant reduction in the RR for NEC (0.23 [95 % CI 0.10–0.55], *p* = 0.0008, Fig. 5a). No effect of *Bifidobacterium breve* in reducing NEC was documented (RR 0.30 [95 % CI 0.01–7.19], *p* = 0.46, Fig. 5b); the only study reporting the use of *Bifidobacterium bifidum* did not report any case on NEC. When the results of studies using *Bifidobacteria* were pooled, a significant reduction in the RR for NEC in the probiotic group was observed (0.24 [95 % CI 0.10–0.54], *p* = 0.0006, Fig. 5c).

*Saccharomyces boulardii* was used in 3 studies [27, 28, 49]: no significant effect of this probiotic was documented (RR 0.81 [95 % CI 0.44–1.49], *p* = 0.50; random effects analysis).

**Fig. 6:**
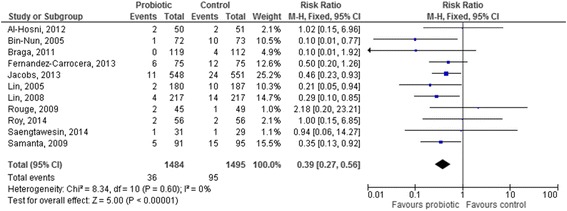
Forest plot showing the association between probiotics and necrotizing enterocolitis in the studies which used a probiotic mix. M-H: Mantel-Haenszel method

The pooled analysis of the 11 studies [26, 32, 33, 35, 37, 38, 40, 41, 48, 50, 58] in which a probiotic mixture was used showed an overall and significant benefit of these products in reducing NEC (RR 0.39 [95 % CI 0.27–0.56], *p* < 0.00001, Fig. 6).

### Study quality

Evaluation of the quality of the studies included in the meta-analysis according to the risk of bias tool as proposed by the Cochrane Collaboration is showed in Table 4, which also shows the level of evidence evaluated following the recommendations of the GRADE Working Group.

**Table 4:** Evaluation of the quality of the studies included in the meta-analysis according to the risk of bias tool as proposed by the Cochrane collaboration and evaluation of the level of evidence according to the GRADE approach

Study	Random sequence generation	Allocation concealment	Blinding	Incomplete outcome data	Selective outome reporting	Other sources of bias	Levels of quality of evidence in the grade approach
Al-Hosni, 2012 [33]	UNCLEAR	UNCLEAR	LOW	UNCLEAR	UNCLEAR	UNCLEAR	LOW
Bin-Nun, 2005 [40]	UNCLEAR	UNCLEAR	HIGH	UNCLEAR	UNCLEAR	UNCLEAR	VERYLOW
Braga, 2011 [35]	LOW	LOW	LOW	LOW	UNCLEAR	LOW	HIGH
Costalos, 2003 [49]	LOW	LOW	LOW	LOW	UNCLEAR	LOW	HIGH
Dani, 2002 [42]	UNCLEAR	LOW	LOW	LOW	UNCLEAR	UNCLEAR	MODERATE
Demirel, 2013 [28]	LOW	LOW	LOW	UNCLEAR	UNCLEAR	UNCLEAR	MODERATE
Dilli, 2015 [44]	LOW	LOW	LOW	UNCLEAR	UNCLEAR	UNCLEAR	MODERATE
Fernández-Carrocera, 2013 [32]	LOW	LOW	LOW	LOW	UNCLEAR	LOW	HIGH
Jacobs, 2013 [26]	LOW	UNCLEAR	LOW	UNCLEAR	UNCLEAR	UNCLEAR	LOW
Kitajima, 1997 [52]	LOW	UNCLEAR	LOW	UNCLEAR	UNCLEAR	LOW	MODERATE
Lin, 2005 [41]	LOW	LOW	LOW	LOW	UNCLEAR	LOW	HIGH
Lin, 2008 [37]	LOW	LOW	LOW	LOW	UNCLEAR	LOW	HIGH
Manzoni, 2006 [37]	LOW	LOW	LOW	UNCLEAR	UNCLEAR	LOW	MODERATE
Mihatsch, 2010 [43]	LOW	UNCLEAR	LOW	LOW	UNCLEAR	LOW	MODERATE
Mohan, 2006 [53]	UNCLEAR	LOW	LOW	UNCLEAR	UNCLEAR	UNCLEAR	LOW
Oncel, 2013 [25]	LOW	UNCLEAR	LOW	LOW	UNCLEAR	UNCLEAR	MODERATE
Patole, 2014 [45]	LOW	LOW	LOW	LOW	UNCLEAR	LOW	HIGH
Rojas, 2012 [30]	LOW	LOW	LOW	LOW	UNCLEAR	LOW	HIGH
Rougé, 2009 [50]	LOW	UNCLEAR	UNCLEAR	UNCLEAR	UNCLEAR	HIGH	LOW
Roy, 2014 [58]	LOW	UNCLEAR	LOW	LOW	UNCLEAR	UNCLEAR	MODERATE
Saengtawesin, 2014 [48]	HIGH	HIGH	HIGH	UNCLEAR	UNCLEAR	UNCLEAR	LOW
Samanta, 2009	LOW	LOW	LOW	UNCLEAR	UNCLEAR	UNCLEAR	MODERATE
Sari, 2011 [34]	LOW	LOW	LOW	UNCLEAR	UNCLEAR	UNCLEAR	MODERATE
Serce, 2013 [27]	LOW	LOW	LOW	UNCLEAR	UNCLEAR	LOW	MODERATE
Stratiki, 2007 [39]	UNCLEAR	UNCLEAR	LOW	UNCLEAR	UNCLEAR	LOW	LOW
Totsu, 2014 [46]	LOW	LOW	LOW	LOW	UNCLEAR	UNCLEAR	MODERATE

### Microbiological quality

Microbiological quality of included studies is described in Table 5. Eight studies were evaluated as having severe microbiological flaws [27, 32, 34, 35, 38, 40, 41, 49], meaning that they did not report a proper probiotic strain identification. Thirteen studies [25, 26, 28, 30, 33, 37, 42–44, 46, 48, 51, 58] were evaluated as having moderate microbiological flaws, because none of them evaluated the probiotic persistence in stools. There were only five studies [39, 45, 50, 52, 53] with minor microbiological flaws.

**Table 5:** Evaluation of the included studies according to their microbiological quality

Author, year	Probiotic strain	Strain identification	Microbiological assessment	Microbiological flaw
Al-Hosni, 2012 [33]	Lactobacillus rhamnosus LGG	LGG identified at the strain level	No assessment	Moderate
	Bifidobacterium infantis	*B. infantis* identified via the web site of the producer: Bifantis (*Bifidobacterium infantis* 35624)		
Bin-Nun, 2005 [40]	Bifidobacterium infantis	Strains not identified at the strain level	No assessment	Severe
	Streptococcus thermophilus			
	Bifidobacterium bifidus			
Braga, 2011 [35]	Lactobacillus casei	Strains non identified clearly	No assessment	Severe
	Bifidobacterium breve			
Costalos, 2003 [49]	Saccharomyces boulardii	Strain not identified at the strain level	*S. boulardii* not characterized in stools.	Severe
			Gut flora assessed by plate count	
Dani, 2002 [42]	Lactobacillus rhamnosus GG	Strain identified	No assessment	Moderate
Demirel, 2013 [28]	Saccharomyces boulardii	Strain identified	No assessment	Moderate
Dilli, 2015 [44]	*B. lactis*	Strain non identified at the strain level but probably Bb12	No assessment	Moderate
Fernández-Carrocera, 2013 [32]	Lactobacillus acidophilus	Strains not identified at the strain level	No assessment	Severe
	Lactobacillus rhamnosus			
	Lactobacillus casei			
	Lactobacillus plantarum			
	Bifidobacterium infantis			
	Streptococcus thermophilus			
Jacobs, 2013 [26]	Bifidobacterium infantis	Strains identified at the strain level	No assessment	Moderate
	Streptococcus thermophilus Bifidobacterium lactis			
Kitajima, 1997 [52]	Bifidobacterium breve	Strain identified	Assessment by a strain-specific monoclonal antibody conjugated with colloidal gold particle	Minor
Lin, 2005 [41]	Lactobacillus acidophilus	Strains not identified at the strain level	No assessment	Severe
	Bifidobacterium infantis			
Lin, 2008 [37]	Lactobacillus acidophilus	Strain identified	No assessment	Moderate
	Bifidobacterium bifidum			
Manzoni, 2006 [51]	Lactobacillus rhamnosus LGG	Strain identified	No assessment	Moderate
Mihatsch, 2010 [43]	Bifidobacterium lactis	Strain identified	No assessment	Moderate
Mohan, 2006 [53]	Bifidobacterium lactis	Strain identified	Species-specific (not strain-specific) assessment	Minor
Oncel, 2013 [25]	Lactobacillus reuteri	Strain identified	No assessment	Moderate
Patole, 2014 [45]	Bifidobacterium breve	Strain identified	Microbiological assessment by PCR	Minor
Rojas, 2012 [30]	Lactobacillus reuteri	Strain identified	No assessment	Moderate
Rougé, 2009 [50]	Bifidobacterium longum	Strain identified	Microbiological assessment by PCR	Minor
	Lactobacillus rhamnosus GG			
Roy, 2014 [58]	Lactobacillus acidophilus, B. longum, B. bifidum, and B. lactis	Strains not identified at the strain level/identification of the commercial product	No assessment	Moderate
Saengtawesin, 2014 [48]	Lactobacillus acidophilus and Bifidobacterium bifidum	Strains not identified at the strain level/identification of the commercial product	No assessment	Moderate
Samanta, 2009	Bifidobacterium infantis	Strains not identified at the strain level	No assessment	Severe
	Bifidobacterium bifidum			
	Bifidobacterium longum			
	Lactobacillus acidophilus			
Sari, 2011 [34]	Lactobacillus sporogenes	Strains not identified at the strain level	No assessment	Severe
Serce, 2013 [27]	Saccharomyces boulardii	Strains not identified at the strain level	No assessment	Severe
Stratiki, 2007 [39]	Bifidobacterium lactis	Strain identified	Assessment by plate count, no strain-specific assessment	Minor
Totsu, 2014 [46]	Bifidobacterium bifidum	Strain identified	No assessment	Moderate

## Discussion

The results of this systematic review and meta-analysis show an overall benefit of probiotic supplementation for the prevention of NEC in preterm infants. These results are strengthened by the absence of significant statistical heterogeneity among studies and by the low-risk of publication bias documented by the funnel plot.

However, despite the overall benefit, it is remarkable that the 26 studies included in the meta-analysis were extremely heterogeneous in terms of probiotic strain, dosage, duration of intervention, and target population. Furthermore, only few studies documented an effective colonization of the infants’ gut with the probiotic strain. Thus, the proposal made by the authors of the recent Cochrane review of a “change in practice” in the use of probiotics in preterm infants [11] might require further investigation.

Currently available literature does not provide any definite conclusion on which probiotic strain should be used, and which group of preterm infants would benefit most from a probiotic intervention. It is important to note that the effect of a live-microorganism used as a probiotic is strictly strain-specific [61]. In this paper we aimed to perform strain-specific sub-meta-analyses but our efforts were weakened by the fact that in very few studies the same probiotic strain was used. For this reason, we were unable to draw definite conclusions on which single-strain of probiotics would be more effective in reducing NEC. When studies using single strains were pooled according to the probiotic *genus*, no significant effect was documented for *Lactobacilli* and *Saccharomyces*. This is partially in contrast with the recent Cochrane review on probiotics and NEC [11], which showed a beneficial effect of *Lactobacilli*: this discrepancy appears to be due mainly to differences in the studies included in the two sub-meta-analyses. Actually, the present meta-analysis included the study by Oncel et al. [25], which was on-going when the Cochrane review was published, but excluded the study by Manzoni et al. [57], where probiotics were used in addition to lactoferrin, and the study by Sari et al. [34], which used a probiotic product which is not properly a *Lactobacillus* [60].

The analysis of studies using *Bifidobacteria* showed a significant effect of *Bifidobacterium breve* in reducing NEC. This is also in contrast with the results of the Cochrane review; however, the discrepancy is explained by the inclusion in the present meta-analysis of the recent study by Dilli et al. [44], which appears to drive the beneficial effect documented for *Bifidobacteria*. Similarly to the Cochrane review [11], the analysis of studies in which more than one strain was used documented a strong and significant effect of these products in the prevention of NEC. No definite conclusion can be drawn from these results, even if it could be suggested that further research should be focused on mixed rather than on single-strain products; a potential rationale for this approach could be that a mix of strains might be more effective in providing an ecological barrier than a single strain.

The evidence that probiotics are effective in reducing NEC in VLBW infants does not necessarily apply also to extremely LBW infants (ELBWs), who are the highest-risk population. Only three studies [26, 33, 58] reported the rate of NEC in ELBWs: in two of these studies [33, 58], the same number of ELBWs in the probiotic and control group developed NEC [33], while in the ProPrems trial NEC incidence was slightly lower in the probiotic group [26]. Given the relatively small number of ELBWs and the inconclusive results, no specific recommendation can be drawn from the analysis of these two studies. Similarly, no study reported separate data for intrauterine-growth-restricted (IUGR) infants, and thus no recommendation can be made either for this high-risk population.

In the analysis of trials evaluating a specific intervention, it is pivotal to understand whether the results of these trials are generalizable or applicable only in specific clinical settings. According to our data, the common belief that probiotics are more effective in populations with a high rate of NEC [62] can be called into question: actually, when studies were divided according to NEC incidence in the control population, NEC reduction was striking and significant also when NEC rate in controls was extremely low. NEC rate in controls can be considered as a proxy for the quality of neonatal care: in this perspective, it is interesting to note that, in contrast with previous data, probiotics appear to confer a preventive benefit also in high quality-of-care settings. NEC rate in control populations was used for the analysis, rather than the baseline NEC rate stated by the authors and used in several studies for sample size calculation: this approach was considered more appropriate, because baseline NEC rate was not provided in many studies and, when provided, there was often a discrepancy with NEC rate detected in controls.

The analysis of included studies according to their microbiological quality points out that clinical studies aiming at evaluate the preventive effect of probiotics on NEC often lack an adequate microbiological assessment and this represents a major limitation of these studies. Actually, it is well known that the correct identification of a probiotic at species level corresponds to evaluate its safety, whereas the identification at strain level is extremely relevant as probiotic beneficial properties are strain-specific. Furthermore, the evaluation of probiotic colonisation, even if temporary, is important to correlate the probiotic presence to the beneficial effects.

The development of gut microbiota in preterm infants is known to be influenced by several factors, including gestational age, mode of delivery, diet, and antibiotic exposure [63]. All these factors are likely to be significant confounders in the relationship between probiotics and NEC: actually, it is well documented that infants fed maternal or donor breast milk have a lower risk of NEC compared to formula-fed infants [64], and that caesarean delivery is associated with a disruption in gut microbiota [65]. Quite surprisingly, however, in published studies data are not analyzed taking these confounders into account [66]. Given the definite protective role of human milk feeding and the symbiotic properties of human milk, it would be fundamental to understand whether the use of probiotics should be encouraged also in human-milk fed infants, or if this intervention should be directed towards exclusively formula-fed infants.

The studies included in the meta-analysis did not report any short-term adverse effect of probiotic supplementation (i.e., bloodstream infection with the probiotic strain). Growing evidence suggests the influence of gut microbiota on long-term health and disease, including both type 1 and type 2 diabetes mellitus, atherosclerosis, asthma, colon cancer, and inflammatory bowel disease [67]. However, at present little is known on the long-term outcome possibly related to the alteration of gut flora in preterm infants, which is the result of the supplementation with exogenous strains.

The choice to investigate a single outcome might be viewed as a limitation of the study: however, this choice was deliberate, as the literature search strategy was focused exclusively on NEC. Any speculation on different outcomes such as sepsis or mortality would have been inevitably misleading, because it would have been impossible to be sure to have identified all the studies reporting on those outcomes.

## Conclusions

Meta-analyses give a valuable contribution in guiding researchers to focus future clinical studies on specific unanswered questions. The results of the present meta-analysis confirm that research on probiotics and NEC is on the right track, but also suggest that there are several unanswered questions which should be addressed before radically changing clinical practice. Our data highlight the need for further, well-designed studies aimed at clarifying the specific effect of probiotics in high-risk populations (i.e., ELBWs, IUGRs) and at addressing the choice of the most effective probiotic product, at the proper dose and duration of supplementation. For this reason, we encourage, for future studies, the publication of study protocols detailing study population and characteristics of the intervention, in order to narrow probiotic research to the most promising strains or combination of strains and to the most vulnerable populations, thus allowing a confirmative individual patient data analysis.
